# Mirolydidae, a new family of Jurassic pamphilioid sawfly (Hymenoptera) highlighting mosaic evolution of lower Hymenoptera

**DOI:** 10.1038/srep43944

**Published:** 2017-03-07

**Authors:** Mei Wang, Alexandr P. Rasnitsyn, Zhongqi Yang, Chungkun Shih, Hongbin Wang, Dong Ren

**Affiliations:** 1 Key Laboratory of Forest Protection, State Forestry Administration, Research Institute of Forest Ecology, Environment and Protection, Chinese Academy of Forestry, 2 Dongxiaofu, Xiangshan Road, Haidian District, Beijing 100091, China; 2 Palaeontological Institute, Russian Academy of Sciences, 123, Profsoyuznaya ul., Moscow 117997, Russia; 3 Department of Palaeontology, Natural History Museum, Cromwell Road, London SW7 5BD, UK; 4 College of Life Sciences, Capital Normal University, 105 Xisanhuanbeilu, Haidian District, Beijing 100048, China; 5 Department of Paleobiology, National Museum of Natural History, Smithsonian Institution, Washington, DC, 20013-7012, USA

## Abstract

We describe Pamphilioidea: Mirolydidae Wang, Rasnitsyn *et* Ren, **fam. n.,** containing *Mirolyda hirta* Wang, Rasnitsyn *et* Ren, **gen. et sp. n.,** from the late Middle Jurassic Jiulongshan Formation of Daohugou, Inner Mongolia, China. The new taxon is characterized by unique forewing venation with the presence of forewing SC, 1-RS almost as long as 1-M, M + Cu straight, 2r-rs strongly reclival, and antenna with homonomous flagellum, revealing new and important details in antennal evolutionary transformations. Thus, *M. hirta* with a combination of primitive and more derived characters highlights its transitional state in the Pamphilioidea and complex mosaic evolution within Pamphilioidea in the late Middle Jurassic. The body of this species is densely covered with thin and long setae, suggesting its possible habit of visiting gymnosperm reproductive organs for pollen feeding and/or pollination during the late Middle Jurassic, much earlier than the appearance of angiosperm flowers.

The small group of distinctive sawflies, Pamphilioidea, has long been considered to comprise four families, viz., two extinct families of Xyelydidae and Praesiricidae and two extant families, Pamphiliidae and Megalodontesidae^1^,^2^,^3^. Over the past decade, phylogenetic analyses on Hymenoptera indicate the monophyly of Pamphilioidea including their two sister extant families^4^,^5^,^6^,^7^,^8^,^9^; however, the relationships of all families in Pamphilioidea were poorly resolved because of limited fossil taxa documented before^5^. Recently, a total-evidence phylogenetic study of Pamphilioidea by combining morphological and DNA sequences data set among extant and extinct species has been conducted^10^. The results suggest that extinct families of Xyelydidae and Praesiricidae are not monophyletic and *Xyelyda* Rasnitsyn (a genus in Xyelydidae) is the sister group to the rest of pamphilioids, while the inter-relationships of Xyelydidae are unresolved. In addition, because members of Praesiricidae together with *Megalodontes* Latreille form a monophyletic group, they proposed that the paraphyletic Praesiricidae to be synonymized under Megalodontesidae^10^.

Pamphilioidea is a small superfamily of distinctive sawflies, characterized by a relatively large head, isolated mandibular foramina and modified ovipositor^1^,^3^. The extinct family Xyelydidae, comprising 12 genera and 35 species distributed in Kyrgyzstan, Siberia, Kazakhstan and China, is the only non-monophyletic family level taxon in Pamphilioidea^1^,^3^,^11^,^12^. Xyelydidae is diagnosed by having the first flagellomere wider and several times longer than the second; forewing vein SC developed and vein R angular or bent near RS base; and the mesopseudosternum distant from fore margin of ventropleuron. However, the external characters of Pamphiliidae are very similar to those of Xyelydidae^13^, except for the synapormorphies of Pamphiliidae: M + Cu bent or angular, sometimes with an additional vein stub at the angulation. Due to the similarity between Xyelydidae and Pamphiliidae, it is difficult to differentiate and classify fossil specimens which might exhibit a mixture of morphological characters. Furthermore, a critical comparison between fossil and extant pamphilioids is lacking, mainly because known fossils are poorly preserved.,

Herein we describe Mirolydidae Wang, Rasnitsyn *et* Ren, **fam. n.**, including one genus and its type species, with distinct characters including antenna, wing venation and legs. The new mirolydid species is based on two specimens collected from the latest Middle Jurassic Jiulongshan Formation at Daohugou Village, Ningcheng County, Inner Mongolia, China. This unique fossil taxon can not be classified into any existing families because it has a striking combination of the unique plesiomorphies and a number of hardly questionable apomorphies in the forewing venation, and special antenna. In our view, this makes it necessary to erect a new family. The discovery of this unique and unusual pamphilioid in the mid Mesozoic is of considerable significance to enhance our understanding of the morphological evolution within the Pamphilioidea or even within the basal Hymenoptera; furthermore, the new data also necessitate a re-evaluation of the evolution of antennal morphology in Pamphilioidea.

## Results

### Systematic Paleontology

Order Hymenoptera Linnaeus, 1758; Superfamily Pamphilioidea Cameron, 1890.

### Family Mirolydidae Wang, Rasnitsyn *et* Ren, fam. n

Type genus. *Mirolyda* Wang, Rasnitsyn *et* Ren, **gen. n.**

#### Diagnosis

Antenna with the composite first flagellomere straight, with basal seven or eight segments connected tightly and inflexibly to each other, in contrast to the following segments interconnected more flexibly; forewing with 1-RS long, almost equal to 1-M in length; 2r-rs strongly reclival; M + Cu straight; legs with hind femora narrow and long; body covered with thin and long setae.

### *Mirolyda* Wang, Rasnitsyn *et* Ren, gen. n

#### Type species of genus

*Mirolyda hirta* Wang, Rasnitsyn *et* Ren, **sp. n.**

#### Etymology

The generic name is a combination of the Latin “Mira-“ meaning strange and unique, and *Lyda*, a junior synonym of *Pamphilius* Latreille, 1802, often used as a suffix for generic names in Pamphilioidea. Gender feminine.

#### Diagnosis

As for family.

### *Mirolyda hirta* Wang, Rasnitsyn *et* Ren, sp. n

#### Material

**Figure 1:**
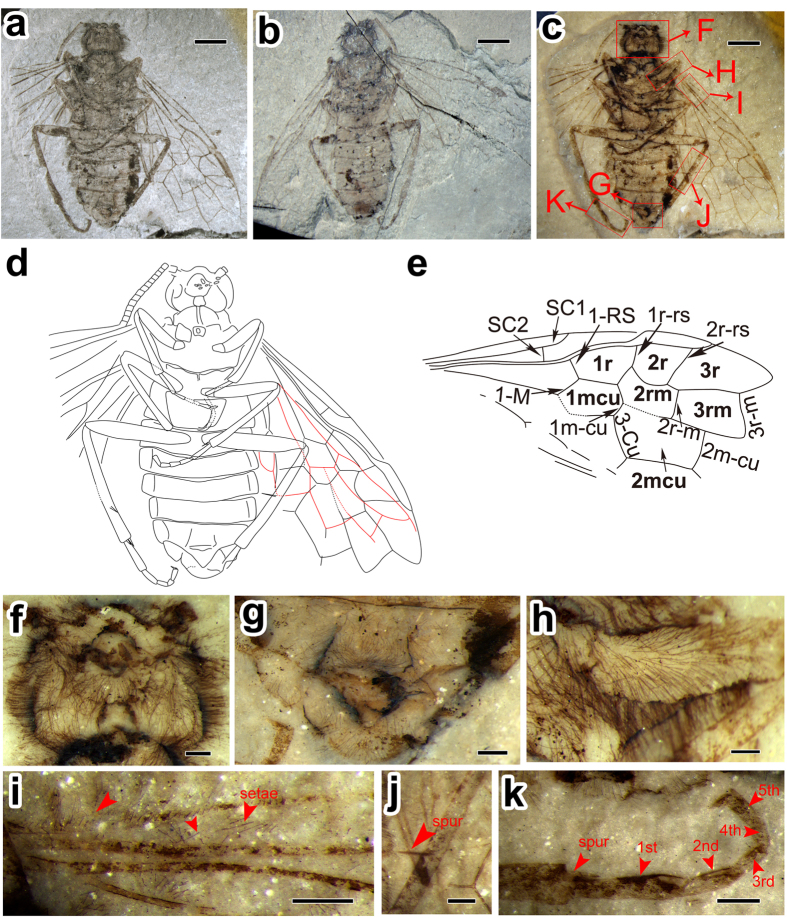
*Mirolyda hirta* gen. et sp. n. Holotype, CNU-HYM-NN2012103 (p/c). (**a**) Photo of part. (**b**) Photo of counterpart. (**c**) Photo of part under alcohol. (**d**) Line drawing of part. (**e**) Line drawing of forewing. (**f**) Head under alcohol. (**g**) Genitals under alcohol. (**h**) Mid femur with long bristles under alcohol. (**i**) Surface of forewing costal area with scattered setae under alcohol. (**j**) Spur on the hind tibia under alcohol. (**k**) Hind tarsi under alcohol. Scale bars: 2 mm in **a**, **b** and **c**; 0.25 mm in **f**, **g** and **h**; 0.5 mm in **i**, **j** and **k**.

**Figure 2:**
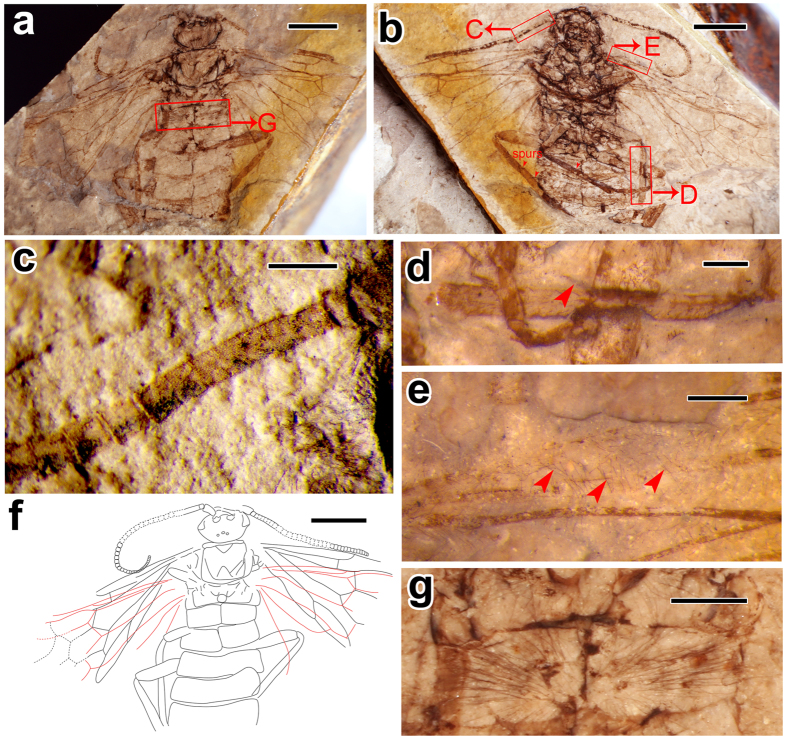
*Mirolyda hirta* gen. et sp. n. Paratype, CNU-HYM-NN2012171 (p/c). (**a**) Photo of part under alcohol. (**b**) Photo of counterpart under alcohol. (**c**) Part of antennal articles under alcohol with oblique illumination. (**d**) Spur on the mid tibia under alcohol. (**e**) Surface of forewing costal area with scattered setae under alcohol. (**f**) Line drawing of part. (**g**) First abdominal segment with long bristles under alcohol. Scale bars: 3 mm in (**a**,**b**) and f; 0.25 mm in (**c**,**d**) and (**e**); 1 mm in (**g**).

Holotype, CNU-HYM-NN2012103 (p/c) and paratype, CNU-HYM-NN2012171 (p/c) at Key Lab of Insect Evolution and Environmental Changes, College of Life Sciences, Capital Normal University (CNUB, Beijing, China) (Figs 1 and 2).

#### Type locality

Jiulongshan Formation, Daohugou Village, Shantou Township, Ningcheng County, Inner Mongolia, China.

#### Type horizon

The latest Middle Jurassic (late Callovian, 165-164 million years ago [Mya])^14^,^15^.

#### Etymology

The specific name is derived from the Latin word “hirtus”, meaning hairy, referring to the thin and long setae covering the entire body.

#### Diagnosis

As for genus.

### Description

Holotype CNU-HYM-NN2012103 (p/c): Male: Part of head, antenna, thorax, abdomen and leg entirely or predominantly dark, pterostigma infuscated; otherwise color pale; whole body densely covered with thin and long setae (those on head sides being up to three times as long as the length of basal flagellomeres); wings with sparse and short setae (Fig. 1c,f–i).

#### Head

Head (Fig. 1f) nearly rounded, about 1.2 times as wide as long; fore margin of clypeus curved and protruding medially; mouth separated from foramen magnum by a bridge; seven or eight basal flagellomeres connected tightly (in contrast to following ones), representing the partially dissociated composite 1st flagellar segment (Fig. 1c,d).

#### Thorax

Prothoracic structure poorly preserved; except that pronotum seems to be very short; mesonotum not distinct; mesopostnotum with round fovea centrally; cenchri long, reaching beyond metanotum midlength; metascutellum rounded triangular, comparatively small; further backward dorsal structures obscure. Mesopseudosternum triangular, well distant from the fore margin of mesoventropleuron.

#### Legs

Legs ordinary; femora fusiform, not particularly wide (hind femur 5.8 times as long as wide); fore and mid femora narrower than the hind one; tibiae comparatively narrow and long (hind tibia 11.3 times as long as wide), covered with dense setae (Fig. 1h), with some preapical and apical spurs preserved; tarsi much shorter than tibia (Fig. 1j); ratio of fore tarsal segments 1:2:3:4:5 = 3.7: 1.6: 1.2: 1: 1.7; ratio of mid tarsal segments 1:2:3:4:5 = 2.6: 1.5: 1.3: 1: 1.3; ratio of hind tarsal segments 1:2:3:4:5 = 5.5: 3.1: 1.8: 1: 2.1 (Fig. 1k).

#### Abdomen

Abdomen slightly wider than mesonotum; terga with no laterotergite separation apparent on dorsal surface; sterna narrow, parallel-sided, laterotergites not wide, with medial margin rounded. Male genitalia insufficiently preserved (Fig. 1g).

#### Wings

Forewing (Fig. 1e) with pterostigma lanceolate, weak and very narrow; the posterior branch of SC subvertical, slightly shorter than 1-RS; R almost straight before RS base and then distinctly bent at RS base; vein 1-RS proclival and relatively long, nearly equal to 1-M in length; M + Cu straight; RS between 1r-rs and 2r-rs arched strongly towards wing posterior margin; 2r-rs inclined basally; 2r-m separated from 2r-rs by 0.43 times of its own length, located distal to middle of cell 2mcu; 3r-m inclined towards wing apex, separated from apex of cell 3r by 0.83 times of its length, and 1.54 times as long as 2r-m; 1m-cu 0.34 times as long as 3-Cu; 2m-cu at middle of cell 3rm. Cell 2r rather small and widened strongly, almost 0.76 times and 0.5 times as long as cells 1r and 3r, respectively; cell 1mcu 1.89 times as long as wide, and as long as cell 2rm; cell 2rm almost 0.85 times as long as 3rm, and 0.66 times as long as and 0.64 times as wide as, cell 2mcu.

Hind wing (Fig. 1d, in red) with SC absent; cell r tapering apically; crossvein 1r-m aligned with 1-M; 1-RS rather long, about 0.59 times as long as 1-M & 1r-m 3r-m separated from apex of cell r by almost its own length; crossvein m-cu slightly longer than 3r-m, joining 2-M distal to midlength of cell rm, separated from 3r-m by a distance equivalent to nearly its length; crossvein cu-a proximal to midlength of cell mcu.

#### Paratype CNU-HYM-NN2012171 (p/c)

Sex unknown. Integument generally brown to dark brown, with scattered setae on whole body, especially, thin and long setae on head, mesothorax and the first abdominal segment (Fig. 2a,b,e and g).

Head (Fig. 2a,e) almost 1.3 times as wide as long; antennal toruli separated as wide as the torular diameter, separated from compound eyes by almost 1.5 times torular diameter; scape 1.85 times as long as maximum wide; pedicel rectangular, 0.86 times as long as maximum wide; antenna long (Fig. 2f), with at least 45 articles preserved; the composite first flagellomere thick and straight, almost 5.1 times as long as wide, with fusion traces of seven inflexibly connected basal flagellomeres as indicated by faint lines when observed under alcohol (Fig. 2c); remaining flagellomeres subquadratic, flexibly connected, tapering distally to form flagellar thread; ocelli small, positioned at posterior part of the head; separated from each other by about their own diameter, separated from the posterior margin of antennal toruli by more than diameter.

Pronotum short, 0.06 times as long as wide; mesoscutum with medial line and notauli strongly impressed; mesoprescutum small, nearly 0.25 times as long as mesonotum; mesoscutellum separated from the mesoprescutum by almost its own length; mesopseudosternum triangular, almost 0.5 times as long as wide, and well distant from the fore margin of mesoventropleuron; metanotum with cenchri small, almost 0.8 times as long as wide, and almost half length of the metanotum; metascutellum rounded quadrate and as long as cenchri. Legs (Fig. 2b) with femora fusiform, hind femur about 5.3 times as long as wide; tibiae comparatively narrow and long, with two preapical spurs preserved, one located almost at the midlength of tibia but the other one distad (Fig. 2b,d); hind tibiae about 0.6 times as wide as hind femora.

Abdomen with five segments preserved; the first one divided medially, covered with long and slender pubescence (Fig. 2g), scattered from the base of first tergum to its periphery.

Forewing (Fig. 2f) with SC two branched, anterior branch of SC meeting C almost at origin of 1-RS, posterior branch vertical, 0.42 times as long as 1-RS; SC + R equal to 1-RS in length; 1-RS with anterior end slightly proximal to its posterior end, and almost as long as 1-M; M + Cu straight; 1cu-a placed proximad middle of cell 1mcu. Cell 1r long, equal to cell 1mcu in length; the latter about 1.85 times as long as wide. Venation of hind wing same as that in holotype.

#### Measurements (in mm)

(Holotype). Body length (excluding antenna) 12.5; head length including mandible 1.83, width (the widest part) 2.3; forewing length up to end of cell 3r 10.7; hind wing length up to the end of cell r 10.5. (Paratype). Body length as preserved 13.3; head length 2.3, width (the widest part) 3.2; forewing length up to end of cell 1mcu 10; hind wing length up to the end of cell r 9.5.

### Phylogenetic analysis

**Figure 3:**
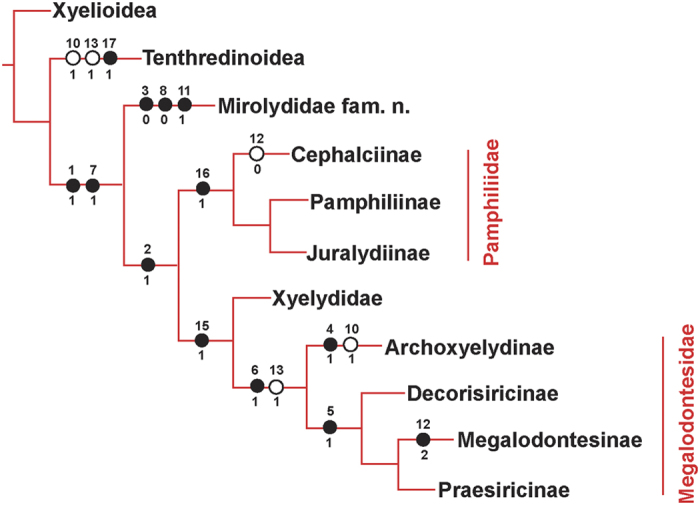
The strict consensus tree of phylogenetic analysis. (●) Nonhomoplastic; (○) homoplastic.

Analysis of the morphological data matrix using NONA yielded only one most parsimonious tree, presented in Fig. 3, with the following characteristics: tree length 26, consistency index (CI) 76 and retention index (RI) 76. The major conclusions of our phylogenetic analysis are as follows: Tenthredinoidea, firstly separated from Xyelioidea, is the sister group to the big branch formed by taxa of superfamily Pamphilioidea. Our target taxon *Mirolyda* **gen. n.** is a sister group to the clade (Pamphiliidae + (Xyelydidae + Megalodontesidae)), supported by the following combination of characters: (i) separated oral and mandibular foramina (character 1, state 1) supporting position of the new genus within the superfamily Pamphilioidea; (ii) forewing with 1-RS not shorter than 1-M (character 8, state 0), supporting its basal position within Pamphilioidea, and (iii) three unambiguous characters indicative of its sister and not ancestral position in respect to the remaining Pamphilioidea: homonomous antenna (character 3, state 0), forewing with 1-RS not shorter than 1-M (character 8, state 0), and forewing with 2r-rs reclival (character 11, state 1), forewing with R distinctly with an angle at RS base (character 7, state 1). Two families of Pamphilioidea, Pamphiliidae and Megalodontesidae, are both recovered as monophyletic with moderate support and with multiple unambiguous morphological characters.

## Discussion

### Mosaic evolution in *Mirolyda hirta* gen. et sp. n.

The affinity of *Mirolyda hirta* **gen. et sp. n**. and Mirolydidae **fam. n.** to superfamily Pamphilioidea is well supported by the following combination of characters: (1) Compact body (without wasp-waist); presence of cenchri; and complete wing venation, especially posterior anal vein present in forewing when observable (excluding Apocrita), (2) Vein RS not furcating (excluding Xyelidae), and (3) Mandibular foramina separated from oral cavity (excluding all Symphyta except for Pamphilioidea).

Nevertheless, *M. hirta* exhibits a unique combination of traits providing new insights into the evolution of pamphilioids. The vein 1-RS of forewing is exceptionally long, almost 0.9 times as long as 1-M, which is a unique plesiomorphic character in the superfamily Pamphilioidea. The new species of *M. hirta* acquires straight M + Cu, similar to that observed in a few megalodontesids^16^ (cf. Taeger *et al*., 2010, pl. 4, Figs 1 and 3) and few xyelydids^10^,^17^ (e.g. *Novalyda cretacica* Gao, Engel, Shih & Ren, *Medilyda* spp.), which is a synapomorphy of Siricoidea s. l. (including Xiphydriidae) + Orussoidea + Apocrita. Besides, 2r-rs is strongly reclival, which is unique in all Symphyta. In addition, the long and extremely thin hind femora of *M. hirta* is unusual and distinctive for Pamphilioidea.

Although the new species *M. hirta* has venation similar to Xyelydidae, it can easily be distinguished from xyelydids by having long 1-RS and straight M + Cu. The length of 1-RS is usually shorter than 1-M in Xyelydidae except that *Rectilyda sticta* possesses 1-RS equal to 1-M in length but aligned linearly with it^12^, not with 1-RS proclival as in *M. hirta*. Besides, straight M + Cu is a diagnostic character for Megalodontesidae excluding Praesiricinae and Rudisiricinae, while M + Cu bent smoothly or angularly in Xyelydidae and Pamphiliidae, respectively.

Permian Parasialidae Ponomarenko, 1977 [Suborder Archimegaloptera Engel, 2004, Order Panmegaloptera Shcherbakov, 2013 (=Megaloptera s.l., i.e. sensu Latreille, 1802)] was considered to be the ancestor of Hymenoptera by Shcherbakov^18^. In contrast, Rasnitsyn presented evidence of the order Palaeomanteida (=Miomoptera) to be a better candidate for the ancestry^2^. Irrespective of their different opinions, wings of Parasialidae and Palaeomanteida (particularly Palaeomanteidae) could be both taken as a rough model of the hymenopteran ancestor, which shows that SC has several fore branches and costal area is very wide, occupying up to 1/4 of wing width. The wide costal area (1/4~1/3 of wing width) is common in other Holometabola, *e.g*., some permotrichopterans and a few mecopterans. The new species of *M. hirta* has a wide costal area almost 1/3 of its wing width, which probably represents the ancestral character state for the Hymenoptera. In summary, unique and exceptional wing structures indicate that *M. hirta* has a combination of primitive and more derived characters highlighting its transitional state in the Pamphilioidea. Such mosaic evolution within Pamphilioidea in the latest Middle Jurassic indicates that the evolutionary process of sawflies is far more complex than we previously thought^19^.

### On the modes of homonomous antenna in Pamphilioidea

**Figure 4:**
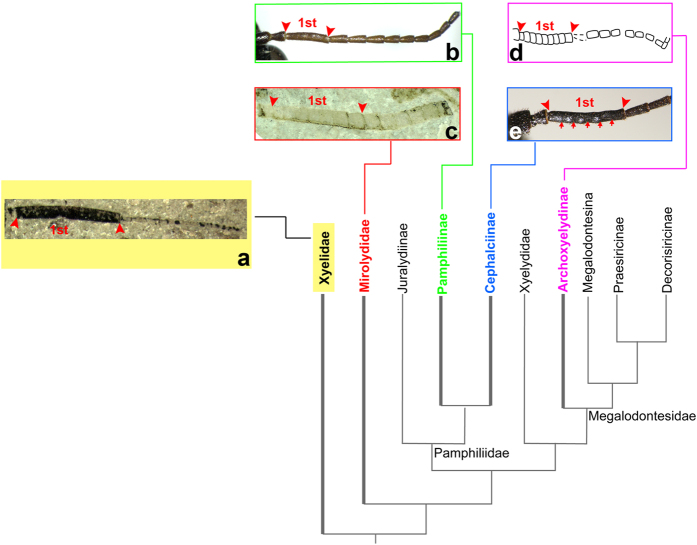
Cladistic relationships of Pamphilioidea modified from Fig. 3, with pattern of the antennae range among Pamphilioidea. (**a**) *Platyxyela* Wang, Shih & Ren (Xyelidae)^36^. (**b**) *Pamphilius* (Pamphiliidae). (**c**) *Mirolyda* gen. n. (Mirolydidae fam. n.). (**d**) *Archoxyelyda* (Praesiricidae)^22^. (**e**) *Caenolyda* (Pamphiliidae).

Given that the antennal diversity of lower Hymenoptera, ranging from the xyelid-like with the composite, thick and long first flagellomere (Fig. 4a) occurring in Xyelidae, Xyelotomidae, and Xyelydidae, etc., to the homonomous antenna in many siricids, tenthredinids, etc, it is widely accepted that the xyelid-like antenna is the plesiomorphic character in Hymenoptera (Table 1). On account of previously accumulated observations of antenna^20^,^21^, Wang *et al*. hypothesized several modes of antennal transformations during the evolution of the order^22^. One of the modes is exemplified by *Archoxyelyda mirabilis* Wang, Rasnitsyn & Ren (Megalodontesidae) that exhibits an extraordinary antenna form with the first nine flagellomeres thick, and tightly connected to each other, in contrast to the following 10–12 segments which are thinner and flexibly connected (Fig. 4d). The first set of thicker and consolidated flagellomeres are interpreted as a possible result of incomplete separation of primary components of the composite first flagellomere in Xyelidae (Fig. 4a).

The new fossils described herein from the Middle Jurassic of China reveal new and important details of the aforementioned scenario of antennal evolutionary transformations. When comparing *Mirolyda* **gen. n.** with *Archoxyelyda*, the holotype of *M. hirta* [CNU-HYM-NN2012103 (p/c)] has the basalmost flagellomeres inflexibly connected, with seven or eight segments combined in a composite first flagellomere (Figs 1c,2c and 3c), while the following segments are flexibly connected. The paratype of CNU-HYM-NN2012171 (p/c) has the basal seven or eight flagellomeres fused and turning to a single long segment as well. The antennal morphology of *Mirolyda* can be interpreted either as an evolutionary stage following the one exemplified by *Archoxyelyda* and consisting of reducing the width of the composite first flagellomere in the *Archoxyelyda*-like antenna. Alternatively, the *Mirolyda*-like antenna might result from direct segmentation of a composite segment that has already become thin, like the one shown in Fig. 4e. Irrespective of how it evolved, we should hypothesize independent acquisition of similar antennal structure by these two genera, because *Mirolyda* with its long 1-RS represents a putatively sister group to all remaining Pamphilioidea, whilst *Archoxyelyda* is rooted deeply within the superfamily^4^, and to hypothesize homology of the antennal structure of the two genera in antennal morphology would result in inferring massive homoplasy in remaining Pamphilioidea.

Traces of the former composite nature of the first flagellomere are known to occur in many living Pamphiliidae^23^. In particular, *Caenolyda* Konow and *Acantholyda* Costa have proceeded the same way of segmentation of the composite first flagellar segment into its component primary segments independently and so form a recent and partial parallel to *Mirolyda* and *Archoxyelyda*. The case of *Caenolyda* and *Acantholyda* is qualified as recent because of their terminal position in the cladogram and missing fossil record^4^. It is unlikely to be homologous with the process in *Mirolyda* and *Archoxyelyda* because the antennae of the latter genera suggest simultaneous segmentation of the composite segment into all primary ones at once, whilst *Caenolyda* and *Acantholyda* demonstrate a rather gradual separation of primary segment one by one starting from the distalmost one.

As a result, it is possible to conclude that our new data highlight existence of at least four modes of restoring homonomous antenna in Pamphilioidea: (i) direct reduction of both length and width of first flagellar segment (*Pamphilius* Latreille, *Neurotoma* Konow, *Megalodontes*); (ii) segmentation of the first segment at once into all primary ones when being wide (*Archoxyelyda*) (Fig. 4d); (iii) same as ii, except that segmentation starts after first segment became as thin as the following segments (*Mirolyda*) (Fig. 4c); (iv) same as ii, but segmentation proceeds by gradual detaching of apical primary segments one by one instead of their simultaneous segmentation (Fig. 4e).

### On the hairy body of *M. hirta* gen. et sp. n.

One of the most interesting characters in *M. hirta* is its pubescent (hairy) body with long and thin setae, which are present but much less in the Xyelidae^24^, e.g. *Chaetoxyela* Rasnitsyn. However, most extant bees often have specialized branched or feathery body setae (hairs) that help them in the collection of pollens^25^. Diverse pubescences often serve different functions, like in bumblebees, thick hairs for insulation, branched hairs for collecting pollen and colored hairs for signals (either warning, or attracting, or both). Moreover, the shape and structure of hairs also differ, like in bees, there are a few compound hairs such as spirally twisted hairs, spatulate hairs, etc^25^. Besides hymenopterans, the Bombyliidae, the so called “bee flies”, one of the largest families in Diptera, are also covered by hairs on their body, and have long, slender legs^26^. The adults of bee flies feed on nectar and pollen, and are believed to be important pollinators of many plants^27^. The new species of *M. hirta* is covered by irregularly scattered setae on the wing membrane as well (Figs 1I and 2e), including a few on the veins, extremely long hairs near the wing base, and on the head, mesothorax and the first abdominal tergum (Figs. 1f and 2g). Besides, the surface structures (setae, small bristles, etc.) on wings have been considered as having close relationships with flight ability in Hymenoptera and also in other related orders^28^,^29^,^30^. Based on aforementioned, it is possible that these hairs of *M. hirta* might have served important and valuable functions such as collecting pollens, providing insulation, sensing the input signals, etc. However, evidence and direct proof are pending.

## Materials and Methods

### Examined taxa and terminologyh

The two type specimens, both with part and counterpart, were collected from a finely laminated tuff in the latest Middle Jurassic Jiulongshan Formation at Daohugou Village, Ningcheng County, Inner Mongolia, China. The Daohugou locality is now considered to be one of the most important insect Lagerstätten^31^,^32^. Because of new calibrations for the Jurassic System, this deposit should be now considered as latest Middle Jurassic (late Callovian) in age^9^, approximately 165 Mya - 164 Mya.

The specimens studied in this paper were examined and then photographed, either dry or wetted with 95% ethanol, with a Leica DFC450 digital camera attached to a Leica M250 C dissecting microscope (Leica, Wetzlar, Germany). The line drawings were prepared using Adobe Illustrator CS2 and Adobe Photoshop CS5 software. The wing venation nomenclature used in this article is modified after Rasnitsyn^1^,^2^. The type materials described are deposited in the Key Lab of Insect Evolution and Environmental Changes, College of Life Sciences, Capital Normal University, in Beijing, China (CNUB; Dong Ren, Curator). Recent species were examined under the dissecting microscopes and kept in the Museum of Forest Biodiversity, Research Institute of Forest Ecology, Environment and Protection, Chinese Academy of Forestry, in Beijing, China (CAF).

### Phylogenetic analysis

A phylogenetic analysis was conducted to elucidate the position of our new family within Pamphilioidea and to clarify familial relationships. In this study, we used the Xyelioidea and Tenthredinoidea as outgroups and nine taxa of superfamily Pamphilioidea as ingroups to carry out the phylogenetic analysis. Eighteen characters were identified and scored for all taxa. The character selection was partly based on the characters used by Ronquist *et al*.^4^, Vilhelmsen *et al*.^6^, and Wang *et al*.^10^ in their phylogenetic analysis of the Pamphilioidea and Symphyta. A complete list of the taxa (Additional file 1:) and the character state matrix (Additional file 2: Table S2) used in the phylogenetic analysis are provided.

The phylogenetic analysis was carried out in NONA^33^ in conjunction with WinClada^34^. Tree searches were performed using an heuristic search method (options: set to hold 10 000 trees, 1000 replications, 100 starting tree replication, multiple TBR + TBR search strategy). Character codings were set up by using Nexus Data Editor 0.5.0^35^ with all characters unordered and of equal weight.

## Additional Information

**How to cite this article:** Wang, M. *et al*. Mirolydidae, a new family of Jurassic pamphilioid sawfly (Hymenoptera) highlighting mosaic evolution of lower Hymenoptera. *Sci. Rep.* **7**, 43944; doi: 10.1038/srep43944 (2017).

**Publisher's note:** Springer Nature remains neutral with regard to jurisdictional claims in published maps and institutional affiliations.

## Supplementary Material

Supplementary Information

## Figures and Tables

**Table 1:** Summary of representative antennae in the lower Hymenoptera.

Family	Antenna type	Length ratio of the composite first flagellomere to the second	Width ratio of the composite first flagellomere to the second
Xyelydidae	xyelid-like	long, at least 3.5 times except for *Ferganolyda* with the first at most as long as the second	almost 1.7 times
Megalodontesidae (except *Megalodontes* and *Archoxyelyda*)	xyelid-like	obviously long, at least 2.6 times	1.4~1.8 times
Pamphiliidae	filiform	short, 1.5~3.5 times, sometimes equal	1~1.6 times
Megalodontesidae (*Megalodontes*)	plumose	1.0~2.3 times	almost equal
Mirolydidae	filiform	5.4~6.2 times	0.9~1 times
Xyelidae	xyelid-like	extremely long, at least 10 times	at least 2.5 times
Tenthredinidae	filiform	1.19~2.25 times	0.7~1 times
Xiphydriidae	filiform	1.6 times	almost equal
Cephidae	filiform	0.94 times	1.1 times
Siricidae	filiform	almost equal	almost equal
