# Supplementary material for: Mirolydidae, a new family of Jurassic pamphilioid sawfly (Hymenoptera) highlighting mosaic evolution of lower Hymenoptera
Source: Sci Rep. 2017 Mar 7;7:43944. doi: 10.1038/srep43944 (PMC5339686; doi:10.1038/srep43944)
Supplement: Supplementary Information [file srep43944-s1.pdf]

SUPPLEMENTARY INFORMATION

Mirolydidae, a new family of Jurassic pamphilioid sawfly (Hymenoptera) highlighting mosaic evolution of lower Hymenoptera

Mei Wang, Alexandr P. Rasnitsyn, Zhongqi Yang, Chungkun Shih, Hongbin Wang[†], Dong Ren[†]

Correspondence: Dong Ren (rendong@cnu.edu.cn); Hongbin Wang (wanghb@caf.ac.cn)

Supplementary Information

Additional file 1: Table S1. Character state matrix of 17 characters for the 11 taxa included in this study.

Additional file 2: Table S2. Definition of 17 characters and their states.

Additional file 1: Table S1. Character state matrix of 17 characters for the 11 taxa included in this study.

	1	2	3	4	5	6	7	8	9	10	11	12	13	14	15	16	17
Xyelioidea (outgroup)	0	0	1	0	0	0	0	0+1	1	0	0	0	2	0+1	0	0	0
Tenthredinoidea (outgroup)	0	0	0+1	0	2	0+1	0	1	0	1	0	1	1	0	0	0	1
Xyelydidae	1	1	1	0	0	0	1	0+1	1	0+1	0	1	2	1	1	0	0+1
Decorisiricinae	1	1	1	0	1	1	1	0+1	1	0	0	1+2	1+2	?	1	0	0
Megalodontesinae	1	1	0+1	0	1	1	0+1	0+1	0+1	0+1	0	2	1+2	?	1	0	0
Praesiricinae	1	1	1	0	1	1	0	1	1	0	0	1	1	?	1	0	0
Archoxyelydinae	1	1	1	1	0	1	1	1	1	1	0	1	1	?	1	0	0
Cephalciinae	1	1	1	0+1	2	0	1	1	2	0	0	0	2	1	0	1	0
Pamphiliinae	1	1	1	0+1	2	0	1	1	2	0	0	1	2	1	0	1	0
Juralydiinae	1	1	1	0	2	0	1	1	2	0+1	0	1	2	?	0	1	0
***Mirolyda* gen. n.**	1	0	0	-	-	0	1	0	0	0	1	1	2	?	?	?	0

Additional file 2: Table S2. Definition of 17 characters and their states.

1. Oral and mandibular foramina: confluent = 0; separated = 1.
2. Mandible: small, neither enlarged nor long and curved = 0; enlarged and curved = 1.
3. Antenna: homonomous = 0; heteronomous= 1.
4. The first flagellomere: obviously without subdivisions= 0; distinctly or just with slight subdivisions = 1.
5. The first flagellomere: several times longer and thicker than the second one= 0; several times as long as, but slightly thicker or not at all thicker, as the second one= 1; varying in length, slightly thicker or not at all thicker, than the second one= 2.
6. Forewing with SC: present= 0; absent= 1.
7. Forewing with R: strongly straight= 0; distinctly with an angle RS base= 1.
8. Length of 1-RS: no shorter than 1-M= 0; much shorter than 1-M= 1.
9. Forewing with M+Cu: strongly straight= 0; bent smoothly= 1; bent angularly, and sometimes with an additional crossvein cu-a or its rudiment= 2.
10. Forewing with 2r-rs: proximal to 2r-m= 0; interstitial or distal to 2r-rs= 1.
11. Forewing with 2r-rs: vertical to R or proximal, strongly or smoothly= 0; reclival= 1.
12. Forewing with cu-a: distinctly distal to midlength of cell 1mcu= 0; at or very close to midlength of cell 1mcu= 1; near base of cell 1cmu= 2.
13. Forewing with cu-a: basal to midlength of cell a=0; just slightly at middle of cell a= 1; near apex of cell a= 2.
14. Nygmata: 1-3= 0; ＞3= 1.

15. 1st abdominal tergite: split medially = 0; not split medially = 1.

16. 2nd abdominal tergite: undivided= 0; divided medially= 1.

17. Forewing with 1-RS: developed= 0; not developed= 1.
